# Digital Health Intervention Combined with Personalized Healthy Breakfast Guidance Improves Breakfast Behavior Among Chinese Young Adults: A Randomized Controlled Trial

**DOI:** 10.3390/nu17203219

**Published:** 2025-10-14

**Authors:** Xinru Wei, Li Huang, Zequn Fu, Qianfeng Liu, Xinyue Yu, Xinrui Zhao, Rong Luo, Feijie Wang, Jiaxin Xiao, Jiayan Xue, Fuzhi Wang, Xingzhao Tian, Shiji Qiu, Meilin Zhang, Huan Liu

**Affiliations:** 1 Department of Nutrition and Food Science, School of Public Health, Tianjin Medical University, Tianjin 300070, Chinazhangmeilin@tmu.edu.cn (M.Z.); 2 Amway (China) Co., Ltd., Guangzhou 510613, China; 3 Nutrilite Health Institute, Shanghai 201203, China; 4 Tianjin Key Laboratory of Environment, Nutrition and Public Health, Tianjin 300070, China; 5 Key Laboratory of Prevention and Control of Major Diseases in the Population, Ministry of Education, Tianjin Medical University, Tianjin 300070, China

**Keywords:** digital health intervention, personalized healthy breakfast guidance, breakfast behavior and body composition

## Abstract

**Objectives**: To evaluate the effects of digital health intervention (DHI) or/and personalized healthy breakfast guidance (PHBG) on the breakfast behavior and body composition of young adults in Tianjin, and to explore the underlying behavioral mechanisms using the Health Action Process Approach (HAPA) framework. **Methods**: In this single-blind, stratified RCT, 160 participants (*n* = 40/group) were randomly assigned to a control group, DHI group, PHBG group, or DHI + PHBG group. Breakfast behavior (primary outcome), HAPA constructs, and body composition were assessed at baseline and after 1 month. Group differences were analyzed using the Kruskal–Wallis test, chi-square test, and linear mixed-effects models. Mediation analysis assessed indirect effects via HAPA variables. **Results**: After a 1-month intervention, adherence to healthy breakfast guidelines was highest in the DHI + PHBG group (80%), followed by the PHBG (72.5%) and DHI (50%) groups, compared to 7.5% in the control group (*χ*^2^ = 51.127, *p* < 0.001, DHI + PHBG group > DHI group: *χ*^2^ = 7.912, *p* < 0.05). All interventions advanced participants along HAPA stages (*H* = 34.678, *p* < 0.001) and improved self-efficacy and planning. PHBG and DHI + PHBG further enhanced outcome expectations, intention, and, for the DHI + PHBG group, self-monitoring. Self-efficacy mediated 17.636% of the PHBG effect and 13.305% of the DHI + PHBG effect, and self-monitoring mediated 7.401% of the DHI + PHBG effect. Waist-to-hip ratios decreased modestly in all intervention groups (*β* = −0.015 to −0.013, *p* < 0.05), but no significant changes were observed in other body composition indices. **Conclusions**: DHI, PHBG, and especially their combination, improved breakfast habits in young adults, with self-efficacy as a key mediator. However, the effects of these interventions on body composition were limited due to the short duration of the trial.

## 1. Introduction

Breakfast is a critical meal for nutrient intake and health outcomes [1]. According to the Chinese Dietary Guidelines (2022), a high-quality breakfast should include at least three of four food groups—cereals/tubers, vegetables/fruits, meat/eggs, and dairy/legumes/nuts. Increased breakfast frequency and quality are linked to improved cognitive performance [2,3,4,5], lower risks of diabetes [6] and cardiovascular disease [7], reduced abdominal obesity [8,9], and better muscle mass maintenance [10]. Despite these benefits, national surveys reveal that most Chinese adults, particularly those aged 18–29, consume unbalanced breakfasts, primarily consisting of cereals and tubers, with over half (55%) eating fewer than three food categories. As young adulthood is a crucial period for establishing long-term dietary habits, effective strategies to improve breakfast quality in this population are urgently needed.

Conventional dietary interventions, such as health education programs, balanced dietary pattern intervention [11], and personalized nutritional guidance [12,13], are increasingly complemented by digital health interventions (DHIs), which leverage digital technologies to improve accessibility, cost-effectiveness, and user engagement [14,15]. Given the widespread smartphone and social media use among young adults, DHIs represent a feasible approach for promoting breakfast improvements [16,17,18,19,20]. Meanwhile, precision nutrition and personalized healthy diet guidance have shown effectiveness in chronic disease management and dietary improvement among middle-aged and elderly populations [21,22,23,24], yet their role in shaping breakfast habits of young Chinese adults remains unclear. No study has examined the separate or combined effects of DHI and personalized healthy breakfast guidance (PHBG) in this population.

Identifying the mechanisms of behavior change is critical for ensuring that newly adopted healthy behaviors are sustained [25,26]. The health action process approach (HAPA), which combines stage and continuity theory [27], provides a framework for understanding the processes of generating and maintaining behavior; specifically, the transition from intention to action. Simultaneously, this model serves as an assessment tool capable of evaluating whether interventions enhance self-efficacy for healthy behaviors, thereby predicting their long-term maintenance. HAPA has been successfully applied to physical activity [28,29], internet use [30], oral health [31], and fruit and vegetable intake [32], making it a valuable tool for elucidating the underlying mechanisms of breakfast behavior change and predicting the long-term effects of behavior intervention approaches in young adults.

Therefore, this study aimed to evaluate the effects of DHI or/and PHBG on breakfast behavior and body composition in young adults aged 18–29 years in Tianjin. Using the HAPA framework, we further sought to identify the mechanisms underlying behavioral change and provide evidence for tailored intervention models to improve breakfast quality and health outcomes in this population.

## 2. Methods

### 2.1. Study Design

The study was a single-blind, stratified, randomized controlled trial conducted for 1 month among young adults from Tianjin in China. Participants were randomly assigned to one of four groups: the digital health intervention (DHI) group, the personalized healthy breakfast guidance (PHBG) group, the combined intervention (DHI + PHBG) group, or the control group. This study was conducted in accordance with the principles of the Declaration of Helsinki and approved by the ethics committee. The trial was registered at the Chinese Clinical Trial Registry (ChiCTR2400084398) on 15 May 2024.

### 2.2. Sample Size and Participants

According to the sample size calculation formula for randomized controlled trial measures, based on reported evidence [33], the anticipated increases in good breakfast behavior after DHI (*p*_1_ = 0.35) and in the control group (*p*_2_ = 0.1), and accounting for a 10% dropout rate, 40 participants were required per group. Prior to enrollment, all participants provided informed consent.

The participants were recruited from May to June in 2024. Initially, 276 individuals were screened for eligibility. Using 1:1 propensity score matching (PSM) based on sex, age (±1 year), education level, and baseline breakfast frequency, 160 participants were selected and included in the trial. These participants, stratified by their baseline HAPA phases, were randomly allocated into four groups: including a control group, a DHI group, a PHBG group, and a DHI + PHBG group (40 participants/group). An independent researcher assigned subjects to groups using randomization codes. The study subjects were not aware of the grouping throughout the process. Personnel responsible for intervention delivery and outcome data collection were unblinded due to the nature of the intervention (i.e., researchers needed to provide guidance). However, outcome assessors responsible for evaluating primary outcomes (such as body composition measurements) were blinded to minimize measurement bias.

According to the Chinese Dietary Guidelines (2022), good breakfast behavior is defined as consuming a nutritious breakfast which contains 4 food categories (cereals/root and tuber crops, meat/eggs, vegetables/fruits, and milk/beans) and the regular consumption of breakfast (≥3 times a week).

The inclusion criteria were young adults (i) aged 18–29 years, (ii) able to comply with the study protocol and sign an informed consent form, (iii) with suboptimal breakfast behavior. Participants meeting all three criteria were included in this study.

The exclusion criteria were (i) pregnancy or lactation, (ii) diagnosis of severe cardiovascular, hepatic, renal, or other systemic diseases, and (iii) any condition potentially interfering with intervention implementation or outcome assessment.

**Figure 1:**
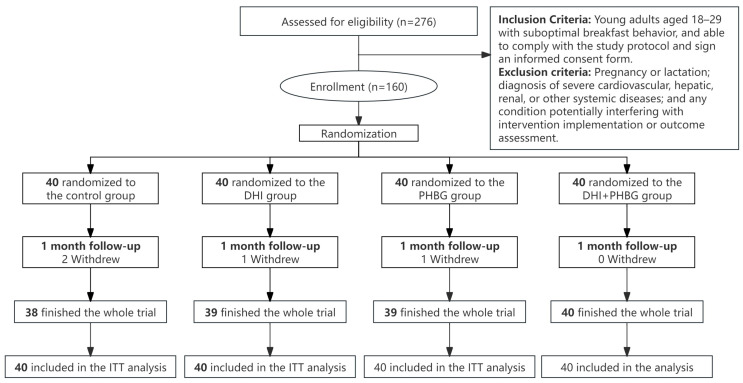
Flowchart of participants.

Eligible participants were stratified by baseline HAPA stage and then randomly allocated to one of the four groups by an independent researcher using computer-generated randomization codes. During the one-month intervention, 4 participants dropped out: 2 (5%) from the control group, 1 (2.5%) each from the DHI and PHBG groups, and none from the combined DHI + PHBG group. All participants were included in the intention-to-treat (ITT) analysis. Multiple imputations were performed for missing data after the intervention. The flow diagram of enrollment, randomization, and follow-up is shown in Figure 1.

### 2.3. Intervention

The intervention lasted for one month. Breakfast energy intake was targeted to account for 25–30% of total daily energy intake. For adults aged 18–29, the recommended breakfast energy range was 425–900 kcal for males and 430–740 kcal for females. Participants were also instructed to maintain their usual dietary structure and energy intake for lunch and dinner. All participants attended weekly “Healthy Breakfast” educational lectures to reinforce awareness of good breakfast practices.

Participants in the control group were asked to keep breakfast records (with photographs) via WeChat but received no personalized guidance or feedback.

Digital Health Intervention (DHI) Group: Participants used the Xiao’an Nutritionist WeChat mini program to record their daily breakfast intake. The WeChat mini program provided automated feedback, including a breakfast quality score and detailed nutrient analysis, based on its built-in dietary tracking and assessment algorithms.

Personalized Healthy Breakfast Guidance (PHBG) Group: This group received customized breakfast plans tailored to their dietary preferences, while adhering to the nutritional principles of the Chinese Dietary Guidelines (2022). These plans emphasized four essential breakfast components (grains, meat/eggs, vegetables/fruits, and milk/beans) and met the breakfast energy needs of healthy adults aged 18–29 years. Participants submitted daily breakfast records (including photographs) via WeChat and received researcher feedback comprising: (i) quality assessments, (ii) food combination suggestions, and (iii) encouragement for maintaining good breakfast habits.

DHI + PHBG Group: Participants received both interventions described above, namely using the Xiao’an Nutritionist WeChat mini program and receiving personalized weekly guidance and feedback from researchers.

Data Collection: At baseline (T0), data were collected on sociodemographic variables, anthropometry, body composition, breakfast behavior assessment, stages, and the social-cognitive variables outlined in the HAPA model. All assessments (except sociodemographic variables) were repeated at the end of the 1-month intervention (T1).

### 2.4. Study Variables

Primary (breakfast behavior) and secondary outcomes (HAPA constructs, anthropometric indices, and body composition) were evaluated at baseline (T0) and post intervention (T1). Sociodemographic data were obtained through interviews using a form developed by the researchers. The questions covered age, sex, education level, and living expenses.

#### 2.4.1. Breakfast Behavior Assessment

The primary outcome was the proportion of participants with good breakfast behavior. Breakfast behavior was assessed using a structured questionnaire. Participants reported the types of foods usually consumed at breakfast, categorized as: (1) cereals/potatoes, (2) vegetables/fruits, (3) meat/eggs, and (4) milk/beans/nuts. They also reported breakfast frequency by answering, “How many times do you have breakfast per week?” These data were used to determine the number of food categories consumed per day and to evaluate overall breakfast behavior. The 24 h dietary recall was used to control the average daily energy intake of the participants at lunch and dinner to remain unchanged from that before the intervention.

#### 2.4.2. Anthropometric and Body Composition Assessment

The participants underwent the following anthropometric measurements: weight and height. Weight was measured using a portable electronic scale with a capacity of up to 150 kg and an accuracy of 100 g, with participants wearing light clothing and no shoes. Height was measured in a standing position using a portable stadiometer, with a range of 200 cm and a variation of 0.1 cm. These values were used to calculate BMI.

For body composition analysis, participants underwent bioelectrical impedance analysis using a tetrapolar device. The following conditions were included in the testing protocol: no pacemaker, water and food fasting (8–12 h), no smoking for at least two hours prior to testing, empty bladder, and no exercise for at least 12 h prior to testing.

#### 2.4.3. HAPA Assessment

Social-cognitive constructs from the HAPA model—including risk perception, outcome expectancies, self-efficacy, intention, planning, perceived social support, and self-monitoring—were evaluated using a validated structured questionnaire. Each construct was measured using multiple items rated on Likert-type scales, with higher scores indicating stronger levels of each psychological factor.

### 2.5. Statistical Analysis

Baseline participant characteristics were described using medians with interquartile ranges (25–75%) for skewed continuous variables and numbers with percentages (%) for categorical variables. The Kruskal–Wallis test and chi-square test were used to compare continuous and categorical variables, respectively. Linear mixed models with repeated measures were used to assess the effects of different intervention patterns, with *β* coefficients and 95% confidence intervals (95% *CIs*) calculated after adjusting for age, sex, education level, and living expenses. Spearman’s rank correlation and conditional logistic regression, adjusted for the same covariates, analyzed associations between HAPA constructs and breakfast quality. Mediation effects of HAPA constructs on breakfast quality were evaluated using SPSS 24.0.

The variables were subjected to the intent-to-treat method, where missing data were imputed using the complete database to obtain postintervention (T1) values from the baseline values of the participants.

All analyses were conducted in SPSS 24.0 with a *p*-value < 0.05 was considered statistically significant.

## 3. Results

### 3.1. Demographics Characteristics of Participants

The baseline demographic and clinical characteristics of participants are summarized in Table 1. No statistically significant differences were observed among the four groups in any baseline variables (all *p* values > 0.05), indicating that the groups were homogeneous and comparable.

### 3.2. Effect of the DHI or/and PHBG on Breakfast Behavior

More than 92% of the participants in both the DHI and DHI + PHBG groups used the Xiao’an Nutritionist WeChat mini program daily. Following the 1-month intervention, all intervention groups demonstrated significant improvements in breakfast behavior. The proportion of participants adhering to good breakfast behavior increased significantly in the DHI (50.0%, *p* < 0.001), PHBG (72.5%, *p* < 0.001), and DHI + PHBG (80.0%, *p* < 0.001) groups compared to baseline. All intervention groups showed significantly higher adherence rates than the control group (*χ*^2^ = 51.127, *p* < 0.001). The number of daily breakfast food categories also increased significantly in all intervention groups (*p* < 0.001). Furthermore, the combined DHI + PHBG intervention led to a significantly greater improvement in good breakfast behavior than the DHI alone group (*χ*^2^ = 7.912, *p* < 0.05). Detailed results are presented in Table 2.

**Figure 2:**
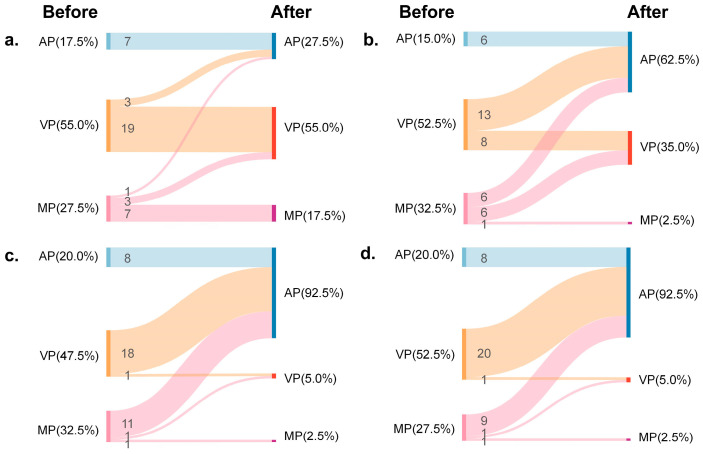
Changes in HAPA phases before and after interventions in different groups. Sankey diagrams illustrating the distribution of participants by group across the HAPA phases (motivation, volition, and action) before and after the intervention. (**a**) Control group, (**b**) DHI group, (**c**) PHBG group, and (**d**) DHI + PHBG group. AP: action phase. VP: volition phase. MP: motivation phase.

After 1 month, no significant phase shifts were observed in the control group (*p* > 0.05). In the DHI group, participants in the action phase increased (from 15.0% to 62.5%, *p* < 0.001), while participants in the motivation phase significantly decreased (from 32.5% to 2.5%, *p* < 0.001). No significant change occurred in the volition phase (*p* > 0.05). In the PHBG group, participants in both the motivation (from 32.5% to 2.5%, *p* < 0.001) and volition phases (from 47.5% to 5.0%, *p* < 0.001) decreased, accompanied by a marked increase in the action phase (from 20.0% to 92.5%, *p* < 0.001). Similarly, in the DHI + PHBG group, the motivation (from 27.5% to 2.5%, *p* < 0.05) and volition phases (from 52.5% to 5.0%, *p* < 0.001) declined, while the action phase rose sharply (from 20.0% to 92.5%, *p* < 0.001). Detailed results are shown in Figure 2.

The Kruskal–Wallis test confirmed that the interventions significantly promoted progression across the HAPA stages (*H* = 34.678, *p* < 0.001).

### 3.3. Effect of DHI or/and PHBG on HAPA Constructs

**Figure 3:**
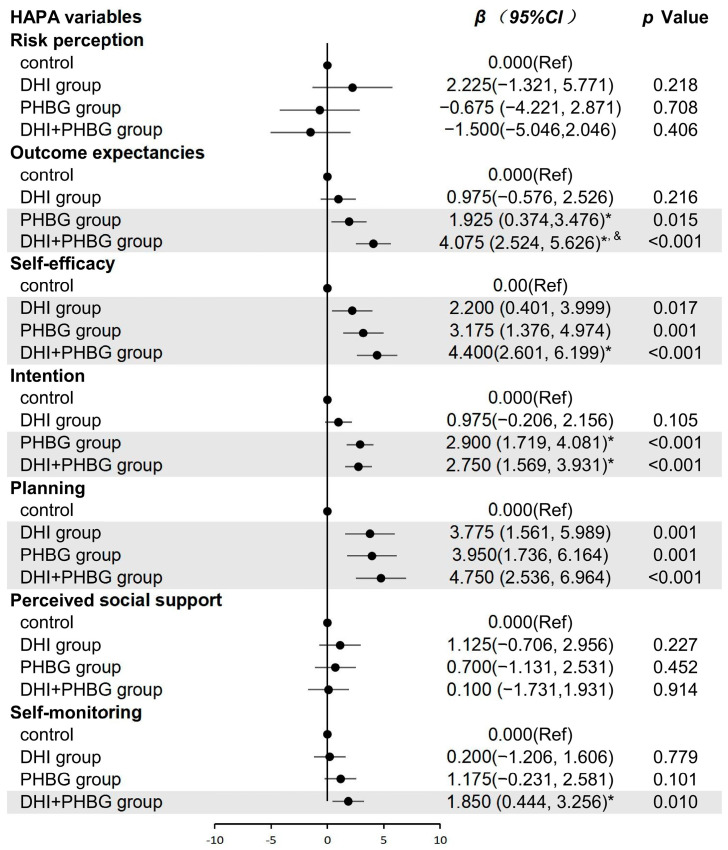
The changes to HAPA constructs after intervention. Model adjusted for sex, age, level of education and living expenses. Control group (*n* = 40) group (*n* = 40), PHBG group (*n* = 40), DHI group (*n* = 40), and DHI + PHBG group (*n* = 40). * *p* < 0.05 compared with baseline and DHI group; ^&^ *p* < 0.05 compared with baseline and PHBG group.

Following the 1-month intervention, significant between-group differences were observed in several HAPA constructs. Compared with the control group, the DHI group showed significant improvements in self-efficacy (*β* = 2.200, 95% *CI*: 0.401, 3.999) and planning (*β* = 3.775, 95% *CI*: 1.561, 5.989). The PHBG group demonstrated significant increases in outcome expectancies (*β* = 1.925, 95% *CI*: 0.374, 3.476), self-efficacy (*β* = 3.175, 95% *CI*: 1.376, 4.974), intention (*β* = 2.900, 95% *CI*: 1.719, 4.081), and planning (*β* = 3.950, 95% *CI*: 1.736, 6.164). The combined DHI + PHBG intervention resulted in significant improvements in outcome expectancies (*β* = 4.075, 95% *CI*: 2.524, 5.626), self-efficacy (*β* = 4.400, 95% *CI*: 2.601, 6.199), intention (*β* = 2.750, 95% *CI*: 1.569, 3.931), planning (*β* = 4.750, 95% *CI*: 2.536, 6.964), and self-monitoring (*β* = 1.850, 95% *CI*: 0.444, 3.256). Detailed results are presented in Figure 3.

### 3.4. Association of the HAPA Constructs and Good Breakfast Behavior

As shown in Table 3, after adjusting covariates, higher self-efficacy (OR:1.443, 95% *CI*: 1.230, 1.692) and higher self-monitoring (OR:1.225, 95% *CI*: 1.051, 1.427) were associated with improved breakfast behavior (*p* < 0.05). Furthermore, Spearman’s rank correlation analyses indicated that outcome expectancies, self-efficacy, intention, planning, perceived social support, and self-monitoring were positively related with good breakfast behavior (*p* < 0.05).

### 3.5. Mediation Analysis of HAPA Constructs on the Intervention–Behavior Association

**Figure 4:**
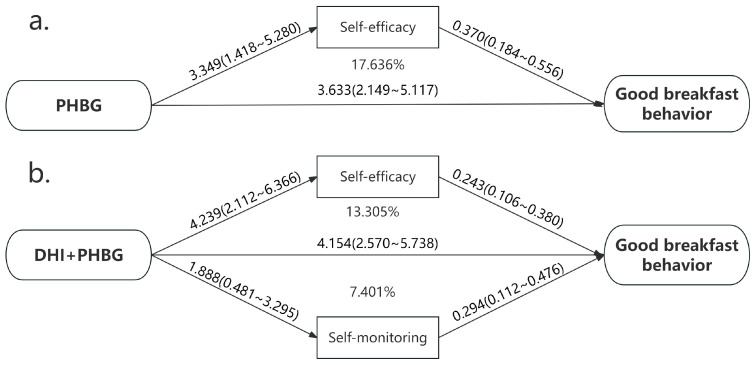
Mediation analysis of HAPA constructs on the association of DHI or/and PHBG and good breakfast behavior. (**a**). Mediated effects of variables in HAPA in association between on PHBG and on good breakfast behavior. (**b**). Mediated effects of variables in HAPA in association between on DHI + PHBG and on good breakfast behavior.

Mediation analysis was performed to examine the potential mediating effects of HAPA constructs on the relationship between the interventions and good breakfast behavior. As illustrated in Figure 4, self-efficacy significantly mediated the effect of the PHBG intervention on breakfast behavior, accounting for 17.636% of the total effect. For the combined DHI + PHBG intervention, both self-efficacy and self-monitoring served as significant mediators, with mediation proportions of 13.305% and 7.401%, respectively.

### 3.6. Effect of the DHI or/and PHBG on Body Composition

Body composition was analyzed under the intent-to-treat principles. A small but statistically significant reduction in waist-to-hip ratio (WHR) was observed in the DHI (*β* = −0.014, 95% *CI*: −0.020, −0.000), PHBG (*β* = −0.013, 95% *CI*: −0.023, −0.005), and DHI + PHBG groups (*β* = −0.015, 95% *CI*: −0.024, −0.005). No other body composition indicators showed significant changes in response to the interventions (BMI, PBF, BMR, SLM, FFM, and SMM, all *p* > 0.05), although all displayed a slight downward trend (Table 4).

## 4. Discussion

This study demonstrated that DHI or/and PHBG interventions improved breakfast behavior by increased adherence to recommended breakfast patterns and greater dietary variety. These interventions also led to notable improvements in key social-cognitive constructs outlined by the HAPA model, including outcome expectations, self-efficacy, intention, planning, and self-monitoring. These findings suggest that PHBG and DHI + PHBG improve breakfast behavior by facilitating transitions across HAPA phases. Furthermore, the DHI or/and PHBG interventions for breakfast reduced the waist-to-hip ratio in young adults.

Digital health tools are increasingly applied to monitor health status and support behavior change [34,35,36]. Mobile health interventions, particularly smartphone applications, provide convenient platforms for self-monitoring, dynamic data collection, and real-time feedback outside clinical settings [37,38]. In this study, the loss-to-follow-up rate in the DHI group was lower than in previous behavioral interventions [39,40], indicating strong acceptability and effectiveness of the breakfast-focused DHI strategy.

Personalized healthy breakfast guidance (PHBG) tailored dietary recommendations and remains resource-intensive yet effective. When combined with digital health intervention (DHI), the integrated approach achieved greater improvements in dietary variety and behavioral stage transitions than either modality alone. Notably, DHI + PHBG outperformed DHI, though no significant difference was observed compared with PHBG. Participants preferred practical, personalized plans tailored to energy needs and preferences, highlighting the irreplaceable role of nutrition professionals. While DHI supports decision-making by providing information and incentives [37], its limited effect may stem from reliance on general feedback without the individualized guidance offered by PHBG.

The HAPA model is widely applied to evaluate health behavior change in areas such as physical activity [32], oral hygiene [41], and diet [42]. Our findings further support its utility in explaining the effect of breakfast interventions. DHI or/and PHBG improved self-efficacy and planning, while PHBG and DHI + PHBG also improved outcome expectations and intentions. DHI + PHBG uniquely enhanced self-monitoring, suggesting stronger potential for long-term behavioral maintenance. Self-efficacy and self-monitoring were the key mediators. DHI + PHBG strengthened confidence and behavioral regulation strategies, helping participants overcome setbacks and sustain healthy habits. In contrast, PHBG primarily enhanced confidence and guided choices through personalized meal plans. Thus, PHBG and DHI + PHBG were effective across motivational and action stages, whereas DHI mainly supported short- and long-term planning but showed weaker sustainability. This limitation may reflect simplified program design and insufficient integration of health behavior theory in app development [43,44]. Future studies should improve engagement by incorporating dynamic updates, personalized reminders [37], and integrating wearable devices [39].

The DHI and/or PHBG interventions also reduced WHR, with trends toward lower BMI, BMR, SLM, and FFM. These findings are consistent with evidence linking high-fiber breakfast patterns, such as fruit, cereal, nuts, and yogurt, to reduced WHR and abdominal obesity [8,45,46,47,48,49]. While participants in the DHI, PHBG, and DHI + PHBG groups experienced a significant reduction in waist-to-hip ratio, the change was minimal (0.01), making it difficult to conclude that the intervention had a meaningful effect on body composition. Body composition is influenced by multiple factors, including diet, exercise, and genetics. Although breakfast behavior improved, changes in diet alone may not be sufficient to cause significant changes in other body composition indicators in the short term. The intervention period in this study was one month, which may be short to induce significant changes in more complex body composition metrics, such as body fat percentage and lean body mass. Future studies with longer intervention durations, potentially incorporating physical activity, are needed to assess the long-term impact of lifestyle interventions on health outcomes.

This study has several strengths. First, by integrating digital health interventions and personalized guidance, it compares the effects of single and combined interventions on dietary behavior, providing a novel, population-tailored nutrition intervention model and laying the foundation for exploring the effects of interventions targeting other meals (such as lunch, dinner, and snacks). This approach could be extended to other meals in future studies. Second, the use of the HAPA model to explain the intervention mechanisms and validate its mediating effects establishes a strong theoretical foundation for broader application of this model in future nutrition research. Third, this study is among the first to explore the impact of breakfast interventions on body composition, contributing to a deeper understanding of the health effects of breakfast behavior.

However, there are several limitations. First, the online recruitment method may lead to a relatively homogeneous sample. Second, supported by previous research, we employed a one-month intervention to assess the primary outcome (behavioral change), which limited the evaluation of the effect of intervention on secondary outcomes (body composition). Third, although total energy intake at lunch and dinner was controlled, the study did not examine the specific dietary patterns during these meals and their potential influence on breakfast behavior and body composition. Finally, given that the participants were young adults rather than a clinical population, although it may have limitations in precision compared to DEXA, the use of bioelectrical impedance analysis (BIA) could a be practical method to measure the body composition in a population study.

Future research should focus on more diverse and larger populations to assess the effectiveness of interventions across different age groups and health conditions. Additionally, this study provides a solid framework for subsequent investigations targeting other meals. Longer-term studies are also needed to evaluate the sustainability of these interventions. To improve control over confounding variables, future research should incorporate more comprehensive dietary monitoring to account for the impact of food or energy intake from other meals on breakfast behavior and body composition. Moreover, more precise methodologies are essential for accurately assessing the impact of interventions on health outcomes.

## 5. Conclusions

DHI or/and PHBG could effectively improve the breakfast behaviors among young people, with the combined intervention of DHI and PHBG superior to DHI alone. Self-efficacy is a key social cognitive factor influencing the behavioral changes in PHBG alone or in combination with digital health intervention. However, the effect of these interventions on body composition remains uncertain. These findings underscore the potential for integrating DHI and PHBG into public health nutrition policies to improve dietary behaviors at a population level. Public health programs should consider adopting multifaceted approaches that combine digital tools with personalized advice to promote sustained, healthy eating habits and improve overall health outcomes in young adults.

## Figures and Tables

**Table 1:** Demographics characteristics of participants in different groups.

Characteristics	Control Group (*n* = 40)	DHI Group (*n* = 40)	PHBG Group (*n* = 40)	DHI + PHBG Group (*n* = 40)	χ^2^/*F*	*p* Value
Sex, *n* (%)					0.202	0.977
Male	18 (45.0)	17 (42.5)	19 (47.5)	18 (45.0)		
Female	22 (55.0)	23 (57.5)	21 (52.5)	22 (55.0)		
Age, years, $\bar{x}$ ± *s*	20.7 ± 2.1	21.2 ± 2.8	21.4 ± 2.5	21.3 ± 2.7	0.613	0.607
Level of education, *n* (%)					0.402	0.94
Senior high school	32 (80.0)	30 (75.0)	32 (80.0)	31 (77.5)		
Undergraduate	8 (20.0)	10 (25.0)	8 (20.0)	9 (22.5)		
Living expenses, *n* (%)					4.638	0.591
≤¥1500/m	12 (30.0)	8 (20.0)	9 (22.5)	12 (30.0)		
¥1500/m~	14 (35.0)	20 (50.0)	17 (42.5)	20 (50.0)		
≥¥2000/m	14 (35.0)	12 (30.0)	14 (35.0)	8 (20.0)		

Results are expressed as mean values ± standard deviation (SD) for ANOVA, and *n* (%) for chi-square test and Fisher exact test. BMI: body mass index.

**Table 2:** Changes in breakfast behaviors before and after the intervention.

Main Outcomes	Group	Baseline	1 Month	χ^2^/*H*	*p* Value
Good breakfast behavior, *n* (%)	Control group	0 (0.0)	3 (7.50)	3.117	0.077
	DHI group	0 (0.0)	20 (50.0) ^#^	26.667	<0.001
	PHBG group	0 (0.0)	29 (72.5) ^#^	45.490	<0.001
	DHI + PHBG group	0 (0.0)	32 (80.0) ^#,^*	53.333	<0.001
Number of breakfast food categories per day, M (P_25_, P_75_)	Control group	2.0 (2.0, 3.0)	3.0 (2.0, 3.0)	1472.000	0.126
	DHI group	2.0 (2.0, 3.0)	3.5 (2.0, 4.0) ^#^	1286.000	<0.001
	PHBG group	2.0 (1.0, 3.0)	4.0 (3.0, 4.0) ^#^	1002.000	<0.001
	DHI + PHBG group	2.0 (2.0, 3.0)	4.0 (4.0, 4.0) ^#,^*	970.500	<0.001

^#^ *p* < 0.05 compared with control group; * *p* < 0.05 compared with DHI group.

**Table 3:** Logistic regression analysis of HAPA variables and good breakfast behavior.

	Model 1		Model 2	
Constructs	OR (95% *CI*)	*p* Value	OR (95% *CI*)	*p* Value
Risk perception	1.075 (1.003, 1.151)	0.042	1.088 (1.009, 1.174)	0.029
Outcome expectancies	1.003 (0.886, 1.135)	0.968	0.984 (0.865, 1.121)	0.813
Self-efficacy	1.428 (1.224, 1.665)	<0.001	1.443 (1.230, 1.692)	<0.001
Intention	1.108 (0.881, 1.394)	0.380	1.069 (0.840, 1.361)	0.585
Planning	0.997 (0.872, 1.139)	0.960	1.012 (0.882, 1.162)	0.864
Perceived social support	1.045 (0.932, 1.170)	0.451	1.038 (0.923, 1.168)	0.530
Self-monitoring	1.225 (1.051, 1.427)	0.009	1.225 (1.051, 1.427)	0.009

Model 1: unadjusted, Model 2: adjusted for age, sex, level of education, and living expenses.

**Table 4:** Changes in body composition indicators.

Indicators	Baseline	1 Month	D-Value	Model 1 *β* (95% *CI*)	*p* Value
BMI (kg·m^−2^)					
Control group	21.67 ± 3.53	21.67 ± 3.43	−0.00 ± 0.48	0 (Ref)	
DHI group	22.00 ± 3.18	21.86 ± 3.35	−0.14 ± 0.58	−0.232 (−0.514, 0.050)	0.106
PHBG group	22.70 ± 3.47	22.56 ± 3.51	−0.14 ± 0.55	−0.141 (−0.422, 0.139)	0.106
DHI + PHBG group	22.11 ± 3.00	21.94 ± 3.02	−0.17 ± 0.56	−0.162 (−0.444, 0.119)	0.258
WHR					
Control group	0.80 ± 0.06	0.81 ± 0.05	0.01 ± 0.03	0 (Ref)	
DHI group	0.81 ± 0.05	0.81 ± 0.05	−0.01 ± 0.01 *	−0.014 (−0.020, −0.000)	0.039
PHBG group	0.82 ± 0.05	0.82 ± 0.05	−0.01 ± 0.02 *	−0.013 (−0.023, −0.005)	0.002
DHI + PHBG group	0.82 ± 0.04	0.81 ± 0.04	−0.00 ± 0.01 *	−0.015 (−0.024, −0.005)	0.002
PBF (%)					
Control group	21.62 ± 8.89	22.00 ± 8.67	0.38 ± 3.58	0 (Ref)	
DHI group	21.65 ± 7.87	21.01 ± 7.82	−0.64 ± 1.82	−1.017 (−2.116, 0.081)	0.069
PHBG group	22.09 ± 8.73	21.89 ± 8.77	−0.20 ± 2.07	−0.572 (−1.671, 0.526)	0.305
DHI + PHBG group	21.19 ± 7.35	20.87 ± 6.77	−0.32 ± 2.08	−0.695 (−1.793, 0.403)	0.213
VFA (cm^2^)					
Control group	47.48 ± 27.17	49.02 ± 25.86	1.54 ± 9.62	0 (Ref)	
DHI group	40.23 ± 25.22	40.46 ± 24.48	0.23 ± 15.06	−1.310 (−6.783, 4.163)	0.637
PHBG group	42.87 ± 29.39	43.07 ± 28.15	0.20 ± 13.17	−1.337 (−6.811, 4.136)	0.630
DHI + PHBG group	39.48 ± 20.40	39.50 ± 20.53	0.01 ± 11.03	−1.525 (−6.998, 3.948)	0.582
TBW (%)					
Control group	33.73 ± 8.26	33.45 ± 8.20	−0.28 ± 1.57	0 (Ref)	
DHI group	35.62 ± 7.95	35.15 ± 7.48	−0.47 ± 2.44	−0.193 (−0.999, 0.614)	0.637
PHBG group	36.24 ± 8.16	35.61 ± 8.10	−0.62 ± 1.96	−0.343 (−1.149, 0.464)	0.402
DHI + PHBG group	35.35 ± 8.18	35.10 ± 7.87	−0.25 ± 1.03	0.032 (−0.774, 0.839)	0.936
BMR (KJ·m^−2^·h^−1^)					
Control group	1364.00 ± 242.83	1356.22 ± 241.69	−7.78 ±44.97	0 (Ref)	
DHI group	1420.75 ± 234.01	1405.75 ± 220.03	−15.00 ± 70.91	−7.225 (−30.644, 16.194)	0.543
PHBG group	1439.22 ± 239.51	1419.95 ± 238.17	−19.27 ± 57.17	−11.500 (−34.919, 11.919)	0.333
DHI + PHBG group	1411.80 ± 240.83	1405.47 ± 231.67	−6.33 ± 30.43	1.450 (−21.969, 24.869)	0.902
SLM (%)					
Control group	43.35 ± 10.64	43.05 ± 10.59	−0.31 ± 1.99	0 (Ref)	
DHI group	45.82 ± 10.28	45.18 ± 9.66	−0.64 ± 3.15	−0.330 (−1.358, 0.698)	0.527
PHBG group	46.62 ± 10.51	45.80 ± 10.45	−0.82 ± 2.48	−0.515 (−1.543, 0.513)	0.324
DHI + PHBG group	45.43 ± 10.54	45.17 ± 10.13	−0.26 ± 1.31	0.045 (−0.983, 1.073)	0.931
FFM (kg)					
Control group	46.04 ± 11.24	45.68 ± 11.18	−0.35 ± 2.07	0 (Ref)	
DHI group	48.66 ± 10.83	47.98 ± 10.19	−0.68 ± 3.28	−0.327 (−1.409, 0.754)	0.550
PHBG group	49.52 ± 11.09	48.63 ± 11.02	−0.89 ± 2.64	−0.540 (−1.622, 0.542)	0.325
DHI + PHBG group	48.26 ± 11.14	47.96 ± 10.74	−0.30 ± 1.41	0.055 (−1.027, 1.137)	0.920
SMM (kg)					
Control group	25.37 ± 6.77	25.27 ± 6.80	−0.10 ± 1.25	0 (Ref)	
DHI group	27.03 ± 6.63	26.52 ± 6.24	−0.51 ± 2.02	−0.405 (−1.054, 0.244)	0.219
PHBG group	27.54 ± 6.73	26.96 ± 6.68	−0.59 ± 1.52	−0.482 (−1.131, 0.166)	0.144
DHI + PHBG group	26.71 ± 6.71	26.62 ± 6.45	−0.09 ± 0.84	0.015 (−0.634, 0.664)	0.963

Model adjusted for sex, age, level of education and living expenses. * *p* < 0.05 compared with control group. WHR = waist-to-hip ratio. PBF = percent body fat. VFA = visceral fat area. TBW = total body water. BMR = basal metabolic rate. SLM = soft lean mass. FFM = fat-free mass. SMM = skeletal muscle mass.

## Data Availability

The data presented in this study are available on request from the corresponding author. The data are not publicly available due to privacy/ethical restrictions.
